# Understanding optimal approaches to patient and caregiver engagement in the development of cancer practice guidelines: a mixed methods study

**DOI:** 10.1186/s12913-017-2107-5

**Published:** 2017-03-09

**Authors:** Melissa C. Brouwers, Marija Vukmirovic, Karen Spithoff, Julie Makarski

**Affiliations:** 1 Department of Oncology, McMaster University, Juravinski Campus, G Wing, 2nd Floor, 1280 Main Street West, Hamilton, Ontario L8S 4L8 Canada; 2 Escarpment Cancer Research Institute, Hamilton, Ontario Canada; 3 Formerly from the Department of Oncology, McMaster University, Hamilton, Ontario Canada

**Keywords:** Patient engagement, Clinical practice guidelines, Cancer, Mixed methods, Consumer engagement

## Abstract

**Background:**

Practice guidelines (PGs) can assist health care practitioners and patients to make decisions about health care options. A key component of high quality PGs is the consideration of patient values and preferences. A mixed methods study was conducted to understand optimal approaches to patient engagement in the development of cancer PGs.

**Methods:**

Cancer patients, survivors, family members and caregivers were recruited from cancer clinics, follow-up clinics, community support programs, a provincial patient and family advisory committee, and a provincial cancer PG development program. Participants attended a workshop, completed a survey, or participated in a telephone interview, to provide information about PG awareness, attitudes, information needs, training, engagement approaches and barriers and facilitators.

**Results:**

Forty-one participants (12 workshop attendees, 21 survey respondents and 8 interviewees) provided data. For those with no PG development experience, fewer than half were previously aware of PGs but perceived several benefits to the inclusion of this perspective. Common barriers to participation across the groups were time commitment, duration of the PG development process, and financial costs. Positive beliefs about the contributions that could be made and practical considerations (e.g., orientation and training, defined roles and expectations) were identified as key features in the successful integration of patients into the PG development process. There was no single model of engagement favored over another.

**Conclusions:**

Study results align with similar studies in other contexts and with international patient engagement efforts. Findings are being used to test new patient engagement models in a programmatic PG development initiative in Ontario, Canada.

**Electronic supplementary material:**

The online version of this article (doi:10.1186/s12913-017-2107-5) contains supplementary material, which is available to authorized users.

## Background

Evidence-based practice guidelines (PGs), defined as systematically developed statements to assist people in making clinical, policy-related and system-related decisions [1, 2], have the capacity to improve health care processes, health system structures, and outcomes of care [3]. One component of a high quality PG is the consideration of the values and preferences of all relevant stakeholders in the development of PG recommendations, including patients or members of the public to whom the recommendations apply [4, 5]. Patient engagement in the PG development process is hypothesized to increase the likelihood that the appropriate questions are asked, the appropriate outcomes are examined, and that patient values and preferences are considered in the interpretation of evidence and in the formulation of recommendations. While international PG development standards include the consideration of patient preferences among the criteria for good quality PGs [4, 5], its integration in the PG development process has been inconsistent, or, in many cases, non-existent [6, 7].

Indeed, while PGs should address issues that are important to patients, and should provide recommendations that align with the range of values and preferences of the target population [8], how best to achieve this is unclear. Recommended methods to identify and integrate patient values into PG development range from consideration of published data on patients’ views, to consultation through the use of patient surveys or focus groups, to active involvement of patients and caregivers as members of PG development groups [9]; however, the evidentiary base supporting these various methods is limited and optimal approaches to engage patients have been poorly studied [10–12]. Moreover, strategies used to recruit patient representatives, extent of patient training and orientation, and methods of engaging patients in the PG development process vary significantly between PG development groups [10].

While efforts are being made internationally to develop patient engagement strategies and toolkits for PG development organizations [9, 13–15], it is unknown whether these resources will be applicable across contexts. In the cancer context, the patient population may be significantly different from patients in other disease settings. People with cancer tend to be older, more frail and vulnerable, sicker, and are often recipients of complex care. These unique factors may make patients’ interest in, or participation in, the PG development process a lower priority than patients whose functionality is higher. For this reason, a study was undertaken to better understand the optimal strategies to engage cancer patients in PG development.

The objective of this study was to obtain information from cancer patients, cancer survivors, and their families/caregivers about their awareness of PGs, attitudes towards PGs, interest in participating in PG development, potential barriers and facilitators to participation, and information needs. The target population included individuals with direct experience as a cancer patient (any diagnosis) or caregiver and who have, and have not had, previous experience as members of PG development groups. The goal is to use this information to advance knowledge about patient engagement in the PG enterprise and to improve the patient engagement process in the cancer PG development initiative in Ontario, Canada.

## Methods

### Context

The Ontario cancer system is overseen by Cancer Care Ontario, an advisory body to the Ontario Ministry of Health, on all matters regarding cancer quality. Cancer Care Ontario provincial programs work with 14 regional cancer programs that are responsible for providing cancer services. In this study, we recruited participants from one regional cancer program, and those involved at a provincial level as members of Cancer Care Ontario’s patient and family advisory council (PFAC) or as existing members of guideline panels with the Program in Evidence-based Care (PEBC), the guidelines program of Cancer Care Ontario. The methods for collecting data were tailored to the preferences of the jurisdictional leaders and the candidate participants themselves and included structured workshops, surveys (paper or electronic modality), and telephone interviews, as described in detail below. Individuals who self-identified as a patient or as a survivor were classified as individuals with direct cancer experience. Individuals who self-identified as a family member or caregiver were classified as individuals with direct caregiver experience.

### Workshops

For the workshops, individuals with direct cancer experience (and caregivers of these individuals) who received care at the Juravinski Hospital and Cancer Centre (JHCC) in Hamilton, Ontario, Canada, or at a cancer support program in this region, were eligible to participate. Participants were recruited by their clinicians or cancer support program leaders and were invited to attend one of three 90-minute workshops, conducted in October 2014. Due to privacy rules, the research team was not provided with a total number of individuals approached. Prior to attending the workshop, participants received an information package that included example PGs (clinician and patient versions), an overview of what is meant by patient engagement in PGs, and a list of additional PG-related resources. At each workshop session, participants were required to read and sign a consent form. The sessions were facilitated by the project manager and a research assistant. Participants were presented with an overview of PGs. This was followed by a facilitated discussion centering on questions related to their awareness of, and attitudes towards, PGs, their interest in participating in PG development, potential barriers and facilitators to their participation, and their information needs. Workshop sessions were audio-recorded, with permission. The recordings were transcribed verbatim and participant codes were used to ensure confidentiality of respondents. Transcripts were independently reviewed by two project members, who conducted inductive and deductive thematic analyses of the data [16].

### Surveys

Eligible participants (those with direct cancer experience or caregiver experience) who were unable or preferred not to attend the regional workshop, were invited to participate by completing a survey (Additional file 1). Staff at JHCC follow-up clinics for breast cancer, colorectal cancer, and prostate cancer, as well as staff at two local cancer support programs, provided the survey package to eligible and interested participants, on behalf of the research team. In addition, the provincial PFAC members at Cancer Care Ontario were invited by the PFAC coordinator, on behalf of the research team, to complete the same survey. Sixty-six survey packages were made available for regional participants and 74 email invitations to Cancer Care Ontario PFAC members were distributed; due to confidentiality rules, we are not able to rule out that individuals may have been approached by both regional recruiters and provincial recruiters. The study materials were the same for both groups.

The survey provided respondents with an overview about clinical PGs, after which they were asked about attitudes towards participating in PG development, anticipated impacts of participation, preferences for participation, guideline information needs, and barriers and enablers to participating. Surveys could be completed on paper or online (LimeSurvey). Response scales followed Likert and Guttman designs. The survey was anonymous and was available for completion from February to April 2015. Participants provided consent with the return of the questionnaire. Quantitative survey data were analyzed using Microsoft Excel.

### Telephone Interviews

Nine individuals who had previous experience contributing to PG development at Cancer Care Ontario’s PEBC were invited to participate in a telephone interview. A research assistant conducted each interview, which was guided by a structured interview form that examined current processes for patients and caregivers in guideline activities (Additional file 2). Upon providing consent, the interviewees were asked to comment on recruitment tactics, training, methods of engaging patients in PG development, actual and potential barriers and facilitators to patient participation, and their personal experience as patient representatives.

Telephone interviews were conducted from February to April 2015. All conversations were audio-recorded, with permission. Interview summary forms were completed after the interview and sent to the interviewees for verification and revision. The forms were then independently reviewed by two project members, who conducted a thematic analysis of the data.

## Results

### Participants

Forty-one people participated in the study. Twelve individuals attended workshops (8 with direct cancer experience, 2 with direct caregiver experience, 1 with both, and 1 unknown), 21 completed surveys (13 regional and 8 provincial; 15 with direct cancer experience, 3 with direct caregiver experience, 1 with both, and 2 unknown), and eight participated in telephone interviews (6 with direct cancer experience, 2 with direct caregiver experience). Twenty-five of the participants (61%) were female. The majority of participants were 50–69 years old (*n* = 25; 61%), had post-secondary degrees or diplomas (*n* = 34; 85%), identified as cancer patients and/or survivors (*n* = 31; 76%), and had direct experience with breast, colorectal or prostate cancer (*n* = 28; 68%). The interview group had a higher proportion of men and a higher proportion of individuals with advanced degrees than did the group who attended a workshop or completed a survey. More detailed information about participant demographics can be found in Table 1.

**Table 1:** Characteristics of participants

Characteristic	Workshop (Regional Cancer Program)	Surveys		Interviews (Provincial Cancer Guideline Program)
		Regional	Provincial	
Total Number	12	13	8	8
Age (years)				
20–29	-	-	-	-
30–39	-	-	1 (12.5%)	-
40-49	2 (16.7%)	1 (7.7%)	2 (25.0%)	-
50-59	3 (25.0%)	3 (23.1%)	3 (37.5%)	4 (50.0%)
60–69	3 (25.0%)	5 (38.5%)	2 (25.0%)	2 (25.0%)
70–79	3 (25.0%)	2 (15.4%)	-	1 (12.5%)
80 +	1 (8.3%)	1 (7.7%)	-	1 (12.5%)
Sex				
Male	4 (33.3%)	2 (15.4%)	4 (50.0%)	5 (62.5%)
Female	8 (66.7%)	10 (76.9%)	4 (50.0%)	3 (37.5%)
Highest education level				
Elementary School	-	-	-	-
High School	2 (16.7%)	3 (25.0%)	1 (12.5%)	-
College Diploma/Degree	6 (50.0%)	6 (50.0%)	1 (12.5%)	1 (12.5%)
Bachelor’s Degree	3 (25.0%)	2 (16.7%)	5 (62.5%)	2 (25.0%)
Master’s Degree or Higher	1 (8.3%)	1 (8.3%)	1 (12.5%)	5 (62.5%)
Current Cancer Experience (multiple answers possible)				
Patient: diagnosed and/or active treatment	1	1	2	1
Survivor: follow-up or survivorship care	9	11	3	6
Caregiver	3	-	4	2
Community Organization Member	4	1	-	3
Type of Cancer ^a^ (multiple answers possible)				
Breast	5	10	1	1
Colorectal	1	1	1	-
Prostate	4	1	-	3
Other	--	-	2	3

^a^Note: This question only applied to participants who identified as patients and/or survivors

### Workshops

Twelve individuals participated in workshops. Participants reported patient personality as a key determinant of whether or not they would join a PG development team. They recommended that primary methods of recruitment should occur through primary care physicians, oncologists or other hospital staff, while patients are receiving their care. It was recommended that patient representatives be involved throughout the PG development process (vs. certain PG development milestones only).

Participants identified potential barriers to patient engagement in the guideline process, including reluctance by patients to share personal cancer experiences with the guideline team (e.g., due to privacy issues, stigma and/or denial), risk of being a tokenized member of the team (vs. a fully integrated member), and the time commitment required to be on the team. In contrast, the belief that patient engagement would positively impact health outcomes for themselves and future patients, and support in favour of their involvement (by their family members, peers and members of the health care team), were identified as facilitators to participation. Further, the provision of training sessions prior to and during involvement in PG development, as well as reimbursement for transportation and other associated costs, were mentioned as additional facilitators to participation.

Prior to attending the workshop, only one participant was aware of the existence of PGs. When probed about their PG information needs, participants agreed that information about prognosis and side effects of treatment, as well as a glossary and list of resources, were important to include in a PG designed for patients. They also advised that PG content be concise, use non-technical language, be presented in a large font, and be available in a variety of formats (e.g., text, bulleted lists, algorithms, electronic applications).

### Surveys

Of the 21 survey participants, 13 were recruited from the regional cancer program and 8 from the provincial program (15% response rate (minimum), assuming no overlap in regional and provincial invitations). There were no differences between the two groups in their views, and so the data were combined. Approximately half of the 21 survey participants (52.4%) were previously aware of PGs and 76.2% reported that it is important for patients to participate in PG development.

While participants reported modest agreement that they would be personally interested in participating in guideline development (mean [m] = 3.19; 5-point scale; 1 = strongly disagree, 5 = strongly agree), they did agree that the impacts of participation could be favorable (Table 2). Most strikingly, they agreed that participation would improve participants’ knowledge about treatment and management options (m = 4.20), promote information sharing (m = 4.14), impact quality of care patients receive (m = 4.10), and impact access to care (m = 4.05).

**Table 2:** Survey participants’ agreement ratings towards participating in practice guideline development, anticipated impacts of participating, and methods of participating (*n* = 21)

Item	Mean^a^	SD
Participating		
I think it is important for me, as a patient/survivor/caregiver, to be involved in practice guideline development.	3.70	1.22
I am interested in participating in practice guideline development.	3.19	1.60
I would want to be provided with training to learn more about practice guidelines as well as the development process, prior to participation.	3.45	1.32
Anticipated Impacts		
By participating in practice guideline development, patients/survivors/caregivers can incorporate their values and preferences into the practice guideline development process.	3.48	1.36
By participating in the practice guideline development process, patients/survivors/caregivers can make suggestions for improvements to the development process.	4.00	0.97
By participating in the practice guideline development process, patients/survivors/caregivers may impact the quality of care patients receive.	4.10	1.05
By participating in the practice guideline development process, patients/survivors/caregivers may impact access to care.	4.05	1.05
By participating in the practice guideline development process, patients/survivors/caregivers may impact the number of available care choices/options.	3.90	1.07
By participating in the practice guideline development process, patients/survivors/caregivers may promote information sharing regarding the disease and related treatment options.	4.14	0.79
By participating in the practice guideline development process, patients/survivors/caregivers may gain valuable knowledge about options available for the treatment and management of cancer.	4.20	0.77
Methods of Participation		
Holding a separate meeting for patients/survivors/caregivers to seek their feedback and to incorporate their values and preferences in the practice guideline report	4.06	0.95
Including patients/survivors/caregivers as members of the practice guideline development group, from start to finish	3.89	1.18
Including patients/survivors/caregivers in the review of a draft version of the practice guideline document only	3.94	1.30
Including patients/survivors/caregivers in communication and information sharing (e.g., participating in the distribution of the final practice guideline document, creating patient versions of practice guidelines)	3.94	1.06

*SD* standard deviation

^**a**^5-point scale (1 = strongly disagree, 5 = strongly agree)

Participants did not show a strong preference for a particular model of patient/survivor/caregiver engagement (Table 2). They did, however, indicate that it was most important for patients to be involved in the creation of the final PG document (76.5%) and in the development of a patient version of the final PG document (94.1%) (Table 3). Participants identified the following information as being most important to include in a PG: benefits and harms of treatment options (95.2%); research evidence supporting the PG recommendations (85.7%); and information regarding the quality of the research that was reviewed (80.9%) (Table 3).

**Table 3:** Survey participants’ perceptions about what information should be in a practice guideline and in what stages of guideline development they should participate

Importance of Information in Practice Guidelines (*n* = 21)		Frequency		
Development Stage	Is it important to include the following in a practice guideline report?	Yes	Maybe	No
* Topic selection*	Information regarding how the practice guideline topic was selected	10 (50.0%)	7 (35.0%)	3 (15.0%)
* Guideline development group*	Information about the doctors, nurses and researchers who were part of the practice guideline development group	10 (50.0%)	4 (20.0%)	6 (30.0%)
* Literature review*	Information on the quality of the research that was reviewed and considered	17 (80.9%)	1 (4.8%)	3 (14.3%)
* Creating a draft document*	Research that supports the various recommendation options considered in the draft practice guideline document	18 (85.7%)	0 (0.0%)	3 (14.3%)
	Information about benefits and harms of the treatment options considered	20 (95.2%)	0 (0.0%)	1 (4.8%)
* Internal and external review*	Information about who was involved in the review of the draft practice guideline document	11 (52.4%)	4 (19.0%)	6 (28.6%)
* Creating a final document*	A summary of how the practice guideline development group made their decisions and reached their final recommendations	14 (66.7%)	5 (23.8%)	2 (9.5%)
Participation in Stages of Practice Guideline Development (*n* = 17)^a^		Frequency		
Development Stage	Is it important for patients to participate in the following stages of practice guideline development?	Yes	Maybe	No
* Topic selection*	The selection of the practice guideline topic	12 (70.6%)	4 (23.5%)	1 (5.9%)
* Literature review*	The collection of research to inform the practice guideline topic	8 (47.1%)	6 (35.3%)	3 (17.6%)
* Creating a draft document*	The development of a practice guideline draft document and its recommendations	7 (41.2%)	6 (35.3%)	4 (23.5%)
* Internal and external review*	The review of a draft practice guideline document and its recommendations	10 (58.8%)	5 (29.4%)	2 (11.8%)
* Creating a final document*	Making changes to a practice guideline draft document based on feedback from the expert review	13 (76.5%)	3 (17.6%)	1 (5.9%)
	The creation of a patient/survivor/caregiver version of the final practice guideline document and its recommendations	16 (94.1%)	1 (5.9%)	0 (0.0%)

^**a**^Four survey respondents indicated that they did not think it is important for patients/ survivors/ caregivers to participate in practice guideline development and therefore did not respond to the “Participation in the Various Stages of Practice Guideline Development” questions above

Lack of available time (m = 3.57), long duration of the PG development process (m = 3.45), and financial costs (m = 3.38), were identified as the largest barriers to guideline participation. Participants reported strongest agreement that facilitators to engagement were personal motivation (i.e. “give back”; m = 4.19), the assumed positive impact of participation on clinical processes (m = 4.19) and the clinical outcomes of others (m = 4.38), and encouragement and support from PG committee members (m = 4.05) and healthcare staff (m = 4.00) (Table 4). They also indicated belief that their care may be positively impacted by participating (m = 4.00).

**Table 4:** Barriers and facilitators associated with participating in the practice guideline development process

Topic	Mean^a^	SD
Barriers (*n* = 21)		
The duration of the practice guideline development process; this can take up to two years	3.45	1.28
Lack of available time to attend practice guideline meetings	3.57	1.25
Financial costs associated with participating (e.g. time away from work, paying for parking)	3.38	1.32
Difficulty finding transportation, to be able to attend practice guideline meetings (e.g. no vehicle, no driver’s license)	2.52	1.47
No/limited access to internet and/or a telephone	2.00	1.10
Current health status (e.g. mobility issues)	2.00	1.10
Lack of knowledge about practice guidelines	2.81	1.21
Lack of technical knowledge (e.g. knowledge about research)	2.50	1.36
Lack of comfort in sharing personal thoughts and experiences within a group setting	2.20	1.20
Lack of confidence in being able to contribute to the practice guideline development process	1.80	0.83
Facilitators (*n* = 21)		
Having a personal interest in the practice guideline topic	3.57	1.12
Having a personal desire to “give back”	4.19	0.75
Knowing that there will be a positive impact on the clinical process, related to the topic of interest	4.19	0.75
Knowing that there will be a positive impact on *your clinical outcomes* (i.e. quality of life; survivorship)	4.00	0.89
Knowing that there will be a positive impact on the *clinical outcomes of others*	4.38	0.74
Being given clear expectations for involvement in the practice guideline development process	3.71	0.85
Receiving *initial* training to support your involvement in the practice guideline development process	3.81	0.81
Receiving *ongoing* training to support your involvement in the practice guideline development process	3.85	0.81
Receiving support/assistance with scientific concepts and terminology	3.81	1.03
Receiving support/encouragement from the nurses, doctors and researchers that make up the practice guideline development team	4.05	0.74
Receiving support/encouragement from the healthcare professionals that provide your care	4.00	0.89
Receiving reimbursement for costs associated with participating (e.g. parking costs)	3.70	1.01

*SD* standard deviation

^**a**^5-point scale (1 = strongly disagree, 5 = strongly agree)

### Telephone interviews

Eight individuals involved in the Cancer Care Ontario PEBC guideline program were interviewed (89% response rate).

- Recruitment to Guideline Panel
  Results from the telephone interviews indicated that recruitment of the eight interviewees to participate in cancer PG development varied: four had been recruited by their health care professionals, two were recruited by PEBC health research methodologists, one was recruited through a support group and one applied to be a patient representative after seeing a public posting. Several interviewees suggested that the best recruiters would be health care professionals (e.g., family physicians, oncologists) because patients trust them and they can target suitable candidates that have a high level of interest in improving patient care and/or the health care system.
  Interviewees commonly stated that the appropriate timing of patient recruitment depends on each patient’s unique situation and personality. All interviewees agreed that, in general, it is not appropriate to actively recruit patients who have been recently diagnosed because they are likely to be overwhelmed with their diagnosis and would not prioritize participation in PG development. In contrast, patients who are receiving follow-up care and selected patients receiving active treatment would be good candidates for recruitment. Having a strong understanding of one’s own and others’ experiences (including caregivers’ experiences) were identified features of a motivated guideline development participant.
- Training
  Six of the eight interview participants did not receive any form of training prior to their involvement as patient representatives. The majority of these six individuals felt that an orientation session to introduce them to PGs and to describe their role as patient representatives would have aided their understanding of the process. Several participants suggested that minimal formal training, if any, would be required to prepare patients for their involvement in PG development and implementation because, as *patient* representatives, their role is to represent the opinions, needs and concerns of other patients, and not to provide medical or technical expertise.
- Scope of Participation
  As members of existing PG committees, four interviewees had contributed to guideline panel discussions, while one had not yet had an opportunity to do so. Two participants were involved in reviewing and editing PG drafts, via mail or email, and had not participated in any in-person meetings. One participant was recently recruited as a patient representative and therefore had not yet had an opportunity to participate in PG development or implementation. Three of the eight interviewees believed that their role on the PG committee reflected their education and career experience more than their experience as a patient or caregiver.
  The majority of participants expressed that patients have the greatest opportunity to contribute to the PG enterprise in a meaningful manner if they are active members of PG committees, as opposed to serving as consultants or only being involved in the communication of the final product. Several interviewees noted the importance of ensuring that patients do not feel isolated from other non-patient members of the committee and that they are provided with an equal opportunity to contribute.
  Participants were asked to comment on the importance of patient participation in the following stages of PG development: topic selection; review of relevant research; creation of a draft PG; review of draft PG; and creation of a final PG document. Participants emphasized the need for patient input on topic selection, creation of a draft PG, and review of the draft copy. Participants felt that the review of relevant research would be too technically complex for the average patient, and that it may be too late to consider patient input during the creation of a final document, as the most important decisions and recommendations are finalized by this point. While some patients may be able to participate in the evidence review and in the creation of a final guideline document, this is dependent on the topic of the guideline and the patients’ individual levels of expertise and skill.
- Barriers and Facilitators to Participation
  A common personal barrier to participating in PG development experienced by the interviewees was the required time commitment, both in terms of preparation and travel. Logistics and capacity related to the document size (which tends to be long), and its technical nature and use of technical language, were seen as additional barriers. Some participants expressed difficulty with feeling connected and engaged in the PG development process.
  Participants agreed that feeling intimidated may be a barrier that prevents patients from participating on PG committees. Intimidation occurs because many non-patient members of these committees are highly trained health care professionals. Therefore, patients may feel uncomfortable commenting or questioning a point being made by one of the committee members. Interviewees agreed that educating patient representatives on *how* to effectively participate on a PG committee would help to address the barrier of intimidation and would effectively support patients in their involvement in the PG development process.
  Common personal motivators that encouraged the interviewees to become involved in PG development included feeling like an equal member of the PG committee, knowing that the contribution of patient representatives improves the patient experience for others, and knowing that patient input is valuable to the project and is appreciated by members of the committee. Additional common facilitators to participation were encouragement and support (from PG committee members, patient and family advisors and members of the PEBC), as well as reimbursement for travel and other associated expenses.
- Participants’ Overall Personal Experiences
  Finally, when asked to comment on their overall experience, some participants expressed feeling underappreciated or being unessential to the PG committee; however, the majority of the interviewees view their participation as patient representatives as a positive opportunity and look forward to remaining involved in PG projects.

## Discussion

Across the three methods (workshops, surveys and interviews) and participant types, there were general consistencies in messages received, particularly as it related to barriers and facilitators to participation. Moreover, these findings align with international efforts and similar patient engagement research in other contexts [9, 10, 17]. Across our methods and participant types, the most commonly identified barriers were time commitment, duration of the PG development process, and financial costs. A feeling of tokenism and reluctance to share personal experiences were also noted. Key facilitators identified by participants were: the belief that participation results in a positive impact on patient health outcomes; having a supportive social environment; orientation/training sessions; and reimbursement for costs associated with participating. Indeed, the findings of this study align with key principles of good governance and effective group management. While tokenism and technicalities related to group projects may be more pronounced for patient participants, many of the issues are common to all groups and participant types. Much has been written about the time, costs, and human resource demands in developing PGs from the perspectives of clinicians and PGs methodologists [18, 19]; there appears to be some common alignment about PG participation as whole, regardless of the stakeholder involved.

This study provided valuable information about cancer patient attitudes towards PGs and the information needs of patients when using PGs to make decisions about their own health care options. Participants did not indicate a clear preference for a particular model of engagement, although those with prior experience in patient engagement activities appeared to prefer active participation throughout the whole PG development process.

One potential concern with our approach is the variation in methods used to collect data and the risk of introducing bias into the results. Indeed, we provided our research partners with considerable latitude to recruit participants in the manner most appropriate for their context. Given the convergence of the data across the different participant types and the different strategies by which they were recruited and participated, we believe there is robustness to our findings. Another limitation of this study is the low survey response rate among the workshop participants and survey participants. While not the purpose of the study, we could not explore differences as a function of participant characteristics (e.g., age, cancer experience [patients vs. survivors vs. caregivers]). Hypothetically, one might be able to tailor the PG experience further to unique clusters of patient characteristics. Similarly, due to the use of convenience sampling methods, participants may not be representative of all potential patient representatives for PG development. Again, however, given that the findings from this study align with previous findings in the literature [10, 15, 17], these concerns may be less of an issue in this particular study. Response rate was high among the telephone interviewees – that is, those with PG experience – and their insights, coupled with PG naive participants and findings from the literature, provide direction for next steps.

Indeed, as a next step to this program of research and to address the findings that emerged, we are currently studying two different models of engagement that vary by modality of participation (traditional vs. remote participation), governance (participants embedded within a guideline group vs. a specific patient/caregiver consultation group), support and training (formal vs. informal), and responsibilities (participation throughout the guideline process vs. participation at discrete periods when perspectives may be most valuable). The results of this study will help identify those features that are most successful at optimizing meaningful participation while mitigating barriers.

## Conclusions

Overall, findings align with international patient engagement efforts and similar studies in PG contexts other than cancer. Key challenges associated with patient engagement include training of patients and guideline panels, group dynamics, and ensuring that patients have reasonable expectations of the impact of their engagement. The study authors are using the results of this study to develop two patient engagement models to test in a programmatic initiative with Cancer Care Ontario and the PEBC in Hamilton, Ontario. This patient engagement study will assess and compare the satisfaction of patient representatives, research staff and PG panel members, with a patient consultation-based model versus an active patient participation model. Results of the pilot test will lead to further optimization of patient engagement in the context of cancer PG development.

## Additional files

Additional file 1: Survey. This survey was completed by a group of eligible participants who were unable or preferred not to attend the workshop sessions. The survey asks respondents to comment on their knowledge of PGs, information and participation preferences, attitudes towards participating in PG development, and anticipated barriers and facilitators to patient and caregiver participation. (DOCX 67 kb)
Additional file 2: Telephone interview guide. This form was used to guide telephone interviews with individuals who had had previous experience contributing to PG development as patients and/or caregivers. The interview form includes questions regarding recruitment tactics, training, methods of engaging patients and caregivers in PG development, barriers and facilitators to participation, as well as questions about their personal experience of contributing to this process. (DOCX 56 kb)

## Abbreviations

JHCC: Juravinski Hospital and Cancer Centre
PEBC: Program in Evidence-Based Care
PFAC: Patient and Family Advisory Council
PG: Practice guideline
