# Supplementary material for: Understanding optimal approaches to patient and caregiver engagement in the development of cancer practice guidelines: a mixed methods study
Source: BMC Health Serv Res. 2017 Mar 9;17:186. doi: 10.1186/s12913-017-2107-5 (PMC5345242; doi:10.1186/s12913-017-2107-5)
Supplement: Additional file 1: — Survey. This survey was completed by a group of eligible participants who were unable or preferred not to attend the workshop sessions. The survey asks respondents to comment on their knowledge of PGs, information and participation preferences, attitudes towards participating in PG development, and anticipated barriers and facilitators to patient and caregiver participation. (DOCX 67 kb) [file 12913_2017_2107_MOESM1_ESM.docx]

**Additional file 1: Survey**

**Patients in the Cancer Guidelines Enterprise:**

**How to Optimize Participation and Meet Information Needs**

**Thank you for your interest and for participating in our research study, *“Patients in the Cancer Guidelines Enterprise: How to optimize participation and meet information needs”* (The PG-PIE Project).**

We thank you for taking the time to contribute to our research work by completing this survey. Your feedback is very important to us. Completing the survey should take no longer than 20-30 minutes.

**Consent to Participate**

Please read through the following information thoroughly to understand your rights as a participant and the objective of this survey. By completing this survey, you are consenting to participate in the PG-PIE study.

**Your Rights as a Participant**

A few important reminders of your rights as a participant in this study’s survey:

- You can withdraw your participation at any time by either (i) not submitting your survey responses or (ii) contacting our office to request that your submitted survey responses be withdrawn (vukmirov@mcmaster.ca).
- You may refuse to answer any questions in the survey.
- Please note that all of your responses are confidential and your name will not be associated with any of your responses at any time (i.e., during data collection, during the analysis of data, during the final presentation of data, etc.).
- We do not foresee significant possible risks or discomforts associated with participating in this research study. However, we would like to bring to your attention that your participation may result in the recollection of your personal experiences with cancer and that those recollections may cause you discomfort. We would like to make you aware of this possibility and kindly suggest that you consider this potential risk in deciding whether or not to participate in our study.
- The PG-PIE Project has received Research Ethics Approval from the Hamilton Integrated Research Ethics Board (HIREB). If you have any concerns regarding your rights as a participant, please contact the HIREB office at 905.521.2100, extension 42680.

**Project Background Information**

***This study is about practice guidelines.***

***Q: What are they?***

A: Practice guidelines are useful documents that can help patients and clinicians make decisions by providing recommendations on the best cancer care options. Practice guidelines are developed by teams of doctors, nurses and researchers. These teams consider research evidence and best practices when creating practice guideline documents. The process of developing *one* practice guideline takes approximately *one to two years*.

***Q: Why is this study being conducted?***

A: In order to develop a high quality and useful practice guideline, it is important to involve patients, survivors and caregivers, to ensure that their opinions and perspectives are considered.

The following are the important questions that we would like to study:

- What do patients, survivors and caregivers know about practice guidelines?
- What are their information needs?
- Do they want to be involved in practice guideline development, and how? *For example, their involvement could include attending practice guideline meetings, taking part in telephone conferences and/or reviewing documents via email or paper-based mail.*

***Q: What is your role in this study?***

A: You will be asked questions about your information needs as a patient, and how other patients, survivors and caregivers may be able to participate in the development of practice guidelines. As a reminder, the process of developing one practice guideline takes approximately one to two years.

**Survey Instructions**

Thank you for agreeing to participate! We welcome your feedback and contributions to our study.

The survey is structured into 5 parts:

Part 1: Current Knowledge of Practice Guidelines

Part 2: Practice Guideline Development

- A: Information Preferences
- B: Participation Preferences
- C: Approaches to Participating

Part 3: Attitudes towards Participating in Practice Guideline Development

Part 4: Barriers and Facilitators to Participating in Practice Guideline Development

Part 5: Demographic Questions

Part 1: Current Knowledge of Practice Guidelines

Practice guidelines are useful documents that can help patients and doctors make decisions by providing recommendations on the best cancer care options. Practice guidelines are developed by teams of doctors, nurses and researchers. These teams consider research evidence and best practices when creating practice guideline documents.

We are interested in learning from you what you know about practice guidelines.

1. Please circle either ‘yes’ or ‘no’ for each of the following questions:

| Before this study, did you know about practice guidelines? | Y N |
| --- | --- |
| Has your doctor ever mentioned a practice guideline when discussing your care? | Y N |
| Have you ever seen a practice guideline? | Y N |
| Have you ever seen a “patient version” of a practice guideline? | Y N |

PART 2. Practice Guideline Development

Please review the following figure. It shows the key steps in practice guideline development and what is involved in each step.

Part 2A. Information Preferences

1. Please rate if you, as a patient/survivor/caregiver, would be interested in having the following information available in a practice guideline.

Circle either ‘yes’, ‘no’ or ‘maybe’ for each statement.

| Practice Guideline Development Stage | **The following is important to include in a practice guideline:** | **Level of Agreement** |
| --- | --- | --- |
| 1.Selecting a topic and bringing together a development team | Information regarding how the practice guideline topic was selected | Yes Maybe No |
|  | Information about the doctors, nurses and researchers who were part of the practice guideline development group | Yes Maybe No |
| 2. Literature Review | Information on the quality of the research that was reviewed and considered | Yes Maybe No |
| 3.Creating a draft document | Research that supports the various recommendation options considered in the draft practice guideline document | Yes Maybe No |
|  | Information about benefits and harms of the treatment options considered | Yes Maybe No |
| 4.Conducting internal and external review | Information about who was involved in the review of the draft practice guideline document | Yes Maybe No |
| 5.Creating a final document | A summary of how the practice guideline development group made their decisions and reached their final recommendations | Yes Maybe No |

1. Do you think that it is important for patients/survivors/caregivers to participate in the development of practice guidelines?

Please circle either ‘yes’ or ‘no’. Yes No

***If you answered ‘yes’, continue onto Part 2B and 2C.**

***If you answered ‘no’, please skip Part 2B and 2C, and go straight to Part 3.**

PART 2B. Participation PREFERENCES (**complete only if you answered ‘yes’ to question 3**)

1. Please rate if you, as a patient/survivor/caregiver, think it is important for patients to participate in the following stages of practice guideline development.

Circle either ‘yes’, ‘no’ or ‘maybe’ for each statement.

| Practice Guideline Development Stage | **It is important for patients/survivors/caregivers to participate in…** | **Level of Agreement** |
| --- | --- | --- |
| 1.Selecting a topic and bringing together a development team | The selection of the practice guideline topic | Yes Maybe No |
| 2.Literature Review | The collection of research to inform the practice guideline topic | Yes Maybe No |
| 3.Creating a draft document | The development of a practice guideline draft document and its recommendations | Yes Maybe No |
| 4.Conducting internal and external review | The review of a draft practice guideline document and its recommendations | Yes Maybe No |
| 5.Creating a final document | Making changes to a practice guideline draft document based on feedback from the expert review | Yes Maybe No |
|  | The creation of a patient/survivor/caregiver version of the final practice guideline document and its recommendations | Yes Maybe No |

PART 2C. Approaches to Participating (**complete only if you answered ‘yes’ to question 3**)

1. This next set of questions asks you to consider the different ways in which patients/survivors/caregivers can be involved with practice guidelines.

Please rate your level of agreement (on a scale of 1-5):

1 = Strongly Disagree 2 = Disagree 3 = No opinion 4 = Agree 5 = Strongly Agree

| **A good way to involve patients/survivors/caregivers in the practice guideline development process is by…** | **Strongly Disagree Strongly Agree** |
| --- | --- |
| Holding a separate meeting for patients/survivors/caregivers to seek their feedback and to incorporate their values and preferences in the practice guideline report | 1 2 3 4 5 |
| Including patients/survivors/caregivers as members of the practice guideline development group, *from start to finish* | 1 2 3 4 5 |
| Including patients/survivors/caregivers in the review of a draft version of the practice guideline document *only* | 1 2 3 4 5 |
| Including patients/survivors/caregivers in communication and information sharing (e.g. participating in the distribution of the final practice guideline document, creating patient versions of practice guidelines) | 1 2 3 4 5 |

PART 3. Attitudes towards Participating in Practice Guideline Development

1. We would like to hear your thoughts about your involvement in the practice guideline development process.

Please rate your level of agreement (on a scale of 1-5):

1 = Strongly Disagree 2 = Disagree 3 = No opinion 4 = Agree 5 = Strongly Agree

| I think it is important for me, as a patient/survivor/caregiver, to be involved in practice guideline development. | Strongly Disagree Strongly Agree    1 2 3 4 5 |
| --- | --- |
| I am interested in participating in practice guideline development. | 1 2 3 4 5 |
| I would want to be provided with training to learn more about practice guidelines as well as the development process, prior to participation. | 1 2 3 4 5 |
| By participating in practice guideline development, patients/survivors/caregivers can incorporate their values and preferences into the practice guideline development process. | 1 2 3 4 5 |
| By participating in the practice guideline development process, patients/survivors/caregivers can make suggestions for improvements to the development process. | 1 2 3 4 5 |
| By participating in the practice guideline development process, patients/survivors/caregivers may impact the quality of care patients receive. | 1 2 3 4 5 |
| By participating in the practice guideline development process, patients/survivors/caregivers may impact access to care. | 1 2 3 4 5 |
| By participating in the practice guideline development process, patients/survivors/caregivers may impact the number of available care choices/options. | 1 2 3 4 5 |
| By participating in the practice guideline development process, patients/survivors/caregivers may promote information sharing regarding the disease and related treatment options. | 1 2 3 4 5 |
| By participating in the practice guideline development process, patients/survivors/caregivers may gain valuable knowledge about options available for the treatment and management of cancer. | 1 2 3 4 5 |

PART 4. Barriers and Facilitators to Participating in Practice Guideline Development

1. Although you may be interested in participating in the practice guideline development process, certain things may make it difficult for you to be involved. Some of these potential barriers are listed below.

Please rate your level of agreement (on a scale of 1-5) that each barrier may prevent you from becoming involved in practice guideline development:

1 = Strongly Disagree 2 = Disagree 3 = No opinion 4 = Agree 5 = Strongly Agree

| The duration of the practice guideline development process; this can take up to two years | Strongly Disagree Strongly Agree    1 2 3 4 5 |
| --- | --- |
| Lack of available time to attend practice guideline meetings | 1 2 3 4 5 |
| Financial costs associated with participating (e.g. time away from work, paying for parking) | 1 2 3 4 5 |
| Difficulty finding transportation, to be able to attend practice guideline meetings (e.g. no vehicle, no driver’s license) | 1 2 3 4 5 |
| No/limited access to internet and/or a telephone | 1 2 3 4 5 |
| Current health status (e.g. mobility issues) | 1 2 3 4 5 |
| Lack of knowledge about practice guidelines | 1 2 3 4 5 |
| Lack of technical knowledge (e.g. knowledge about research) | 1 2 3 4 5 |
| Lack of comfort in sharing personal thoughts and experiences within a group setting | 1 2 3 4 5 |
| Lack of confidence in being able to contribute to the practice guideline development process | 1 2 3 4 5 |

Please describe any other things that would discourage you from participating in practice guideline development, in the box provided below.

1. On the other hand, there may be some things that would support or encourage your participation in the practice guideline development process.

Please rate your level of agreement (on a scale of 1-5) that each of the following may support your involvement in practice guideline development:

1 = Strongly Disagree 2 = Disagree 3 = No opinion 4 = Agree 5 = Strongly Agree

| Having a personal interest in the practice guideline topic | Strongly Disagree Strongly Agree    1 2 3 4 5 |
| --- | --- |
| Having a personal desire to “give back” | 1 2 3 4 5 |
| Knowing that there will be a positive impact on the clinical process, related to the topic of interest | 1 2 3 4 5 |
| Knowing that there will be a positive impact on *your* *clinical outcomes* (i.e. quality of life; survivorship) | 1 2 3 4 5 |
| Knowing that there will be a positive impact on the *clinical outcomes of others* | 1 2 3 4 5 |
| Being given clear expectations for involvement in the practice guideline development process | 1 2 3 4 5 |
| Receiving *initial* training to support your involvement in the practice guideline development process | 1 2 3 4 5 |
| Receiving *ongoing* training to support your involvement in the practice guideline development process | 1 2 3 4 5 |
| Receiving support/assistance with scientific concepts and terminology | 1 2 3 4 5 |
| Receiving support/encouragement from the nurses, doctors and researchers that make up the practice guideline development team | 1 2 3 4 5 |
| Receiving support/encouragement from the healthcare professionals that provide your care | 1 2 3 4 5 |
| Receiving reimbursement for costs associated with participating (e.g. parking costs) | 1 2 3 4 5 |

Please describe any other things that would encourage you to participate in practice guideline development, in the box provided below.

Part 5: Demographic Questions

This is the final section of the survey. The answers to these questions will help us to understand the characteristics of our participants and to summarize the information we receive from the surveys.

1. **Gender**:  Male  Female
2. **Age**:  18-29  30-39  40-49  50-59  60-69  70-79  80+
3. **City/Town**: _____________________________________
4. **Highest education level** (multiple answers possible):

1. **Current cancer experience** (multiple answers possible):

1. **Type of cancer – if applicable** (e.g., breast, colorectal, lung, etc.)

_____________________________________

After having participated in this survey, would you like to learn more about practice guidelines? Y N

The survey is now complete.

**Thank you for participating!**
