# Supplementary material for: Understanding optimal approaches to patient and caregiver engagement in the development of cancer practice guidelines: a mixed methods study
Source: BMC Health Serv Res. 2017 Mar 9;17:186. doi: 10.1186/s12913-017-2107-5 (PMC5345242; doi:10.1186/s12913-017-2107-5)
Supplement: Additional file 2: — Telephone interview guide. This form was used to guide telephone interviews with individuals who had had previous experience contributing to PG development as patients and/or caregivers. The interview form includes questions regarding recruitment tactics, training, methods of engaging patients and caregivers in PG development, barriers and facilitators to participation, as well as questions about their personal experience of contributing to this process. (DOCX 56 kb) [file 12913_2017_2107_MOESM2_ESM.docx]

**Additional file 2: Telephone interview guide**

**Patients in the Cancer Guidelines Enterprise:**

**How to Optimize Participation and Meet Information Needs**

**Thank you for your interest and for participating in our research study, *“Patients in the Cancer Guidelines Enterprise: How to optimize participation and meet information needs”* (The PG-PIE Project).**

We thank you for taking the time to contribute to our research work by participating in this interview. Your feedback is very important to us.

**Project Background Information**

The PG-PIE study is about practice guidelines. Current research suggests that patient involvement is a key component of a high-quality practice guideline. Including patients/survivors/caregivers throughout the practice guideline development process helps to create a more useful final practice guideline document.

However, there are several unanswered questions regarding patient involvement that we would like to study. We would like to find out:

- If patients know about practice guidelines
- What their information needs (regarding practice guidelines) are
- If they are interested in becoming involved in practice guideline development, and if so, how they would like to be involved

As a **PEBC patient representative**, you will be asked questions about your experience as a patient representative, and about your opinions regarding patient/survivor/caregiver participation in practice guideline development.

**Demographic Information**

To begin the survey, I would like to ask a few demographic-related questions. This information will help the project team to understand the characteristics of the interviewees.

1. **Gender**:  Male  Female
2. **Age**:  18-29  30-39  40-49  50-59  60-69  70-79  80+
3. **City/Town**: _____________________________________
4. **Highest education level** (multiple answers possible):

1. **Current cancer experience** (multiple answers possible):

1. **Type of cancer** (if applicable) ________________________________

Part 1: Recruitment

1. a) How were you recruited to participate in the PEBC, and by whom?

b) Do you believe this way to recruit participants is the best? Are there any modifications that could be made to current tactics, or any other strategies you would recommend?

1. Three types of individuals will be recruited to participate in practice guideline development: patients (undergoing active treatment), survivors (undergoing follow-up care), and caregivers.

For individuals who have/or have had cancer, at what stage of their cancer journey do you think it would be best to involve them in practice guideline development?

*For example:*

- *when they are first diagnosed*
- *while they are undergoing active treatment*
- *once their active treatment is complete and they are undergoing survivorship care*

Part 2: Training

1. a) Did you receive any form of training to prepare you for your involvement with the PEBC? Do you feel as though it was sufficient?

b) In practice guideline development, what type of training do you think would be required to adequately prepare participants to do this sort of work?

Part 3: Participation

1. How are/were you involved in the PEBC? What particular aspects of practice guideline development are/were you involved in?
2. Over the course of one to two years, the practice guideline development team works through five key stages of development.

Do you think it is important for patients, survivors and caregivers to participate in the following stages of guideline development? Why or why not?

| **Stage** | **Comments** |
| --- | --- |
| Topic Selection |  |
| Review of the relevant research |  |
| Creation of a draft practice guideline document |  |
| Review of the draft document |  |
| Editing, and creation of a final practice guideline document |  |

1. There are many models one could follow to involve patients, survivors and caregivers in the development of practice guidelines. For example:

- Patients/survivors/caregivers can actively *participate* in practice guideline development, for example, by becoming members of the guideline development team and becoming involved in either some, or all, stages of practice guideline development.
- They can be *consulted* by the guideline development team. This requires less involvement than active participation. For example, consultation would involve collecting information from participants to inform practice guideline development. Data collection would likely involve the use of surveys, interviews, focus groups, etc.
- Finally, they can become involved in the *communication* of the final practice guideline recommendations (e.g. by disseminating practice guidelines to patients).

What are your opinions about the three models of involvement? Is one better than the other?

Part 4: Barriers and Facilitators to Participation

1. Although patients/survivors/caregivers may show an interest in participating in practice guideline development, certain things might reduce their interest, or prevent their ability to participate *(e.g. transportation, reluctance to share private experiences, lack of technical knowledge, etc.)*
2. Did anything make it hard for you to participate? If so, how did you overcome the challenges that you experienced?
3. Can you think of any other things that maybe you, yourself, did not experience, but you think might prevent others from participating in practice guideline development?
4. On the other hand, some things could help to encourage or to support participation *(e.g. incentives such as reimbursement for travel costs, training, sense of “giving back”, support from the patient healthcare team, etc.)*
5. What encouraged you to participate? Were there any things that were done to make it easier for you to participate?
6. Can you think of any additional things that might encourage others to participate in practice guideline development?

Part 5: Additional Notes

1. Before we end the interview, is there anything else that you would like to share about your PEBC experience?
